# Superior mesenteric artery syndrome in an 8-year-old boy: a case report

**DOI:** 10.1186/s13256-023-04061-2

**Published:** 2023-08-12

**Authors:** Ayah Aldagher, Rodaina Almasri, Jaber Mahmoud

**Affiliations:** 1 Faculty of Medicine, Damascus University, Damascus, Syria; 2 Division of Gastroenterology, Pediatric University Hospital, Damascus, Syria

**Keywords:** Superior mesenteric artery syndrome, Wilkie's syndrome, SMAS, Case report, Duodenojejunal anastomosis

## Abstract

**Background:**

Superior mesenteric artery syndrome (SMAS), also known as Wilkie's syndrome, is a rare disease that mainly presents as intestinal obstruction with a variable incidence ranging from 0.013 to 0.3% (Salehzadeh et al. in Case Rep Gastrointest Med, 2019, 10.1155/2019/3458601). In this syndrome, the third part of the duodenum, located between the abdominal aorta and the superior mesenteric artery, is wholly or partially obstructed (Mosalli et al. in J Pediatr Surg 46:e29–31, 2011).

**Case presentation**

An 8-years-old Arabian male patient was admitted to the gastrointestinal department at the pediatric hospital in Damascus, Syria, with complaints of chronic abdominal pain and periodic vomiting since he was two years old. At the age of Seven, he was diagnosed with appendicitis, and after two months of persistent symptoms, he was diagnosed with an umbilical hernia. Finally, after a long time of suffering, he was diagnosed with SMAS and underwent a successful surgical operation. After 3 months of follow-up, he was in good health with no symptoms.

**Conclusion:**

Whenever a patient complains of vomiting and chronic abdominal pain, intestinal obstruction is suspected, Common differential diagnoses were excluded and the cause is anonymous, we should consider superior mesenteric artery syndrome.

## Background

Superior mesenteric artery syndrome (SMAS), also known as Wilkie's syndrome, is a rare disease that mainly presents as intestinal obstruction with a variable incidence ranging from 0.013 to 0.3% [1] In this syndrome, the third part of the duodenum, located between the abdominal aorta and the superior mesenteric artery, is wholly or partially obstructed [2]. Due to the lack of retroperitoneal and visceral fats, the superior mesenteric artery and abdominal aorta typically form an angle of 6° to 25° instead of the normal angle of 38° to 56°, which causes this rare, life-threatening syndrome [3]. The symptoms typically include chronic abdominal pain, nausea, early satiety, loss of appetite, and vomiting [4]. Patients typically suffer from SMAS after rapid weight loss or spinal surgery. Therefore, SMA syndrome is unexpected among young adults who lack these traditional risk factors [5]. The management of SMA syndrome can be conservative or surgical [6].

## Case presentation

**Fig. 1:**
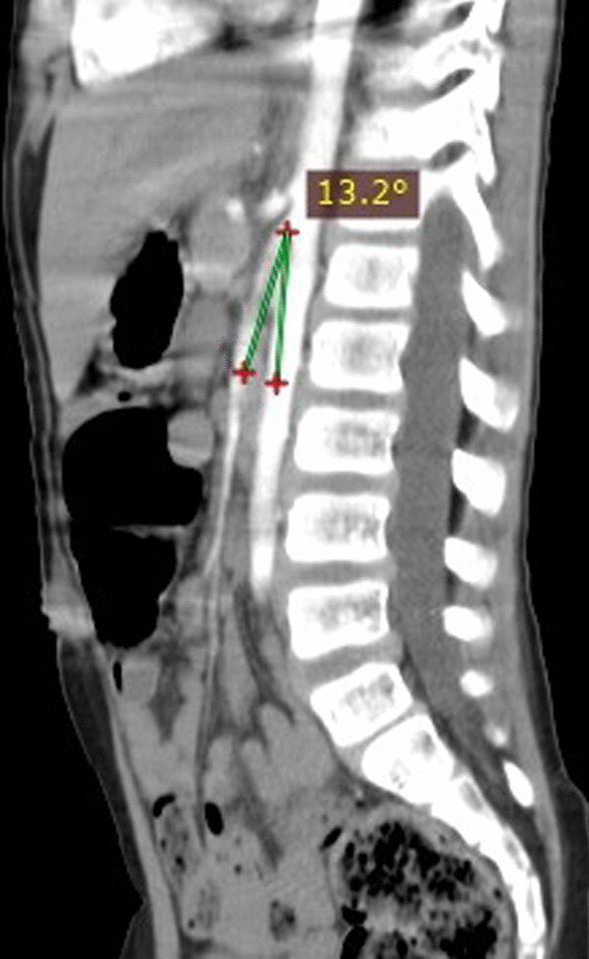
Contrast enhanced CT scan abdomen and pelvis (sagittal view) showing the angle between the abdominal aorta and the superior mesenteric artery

**Fig. 2:**
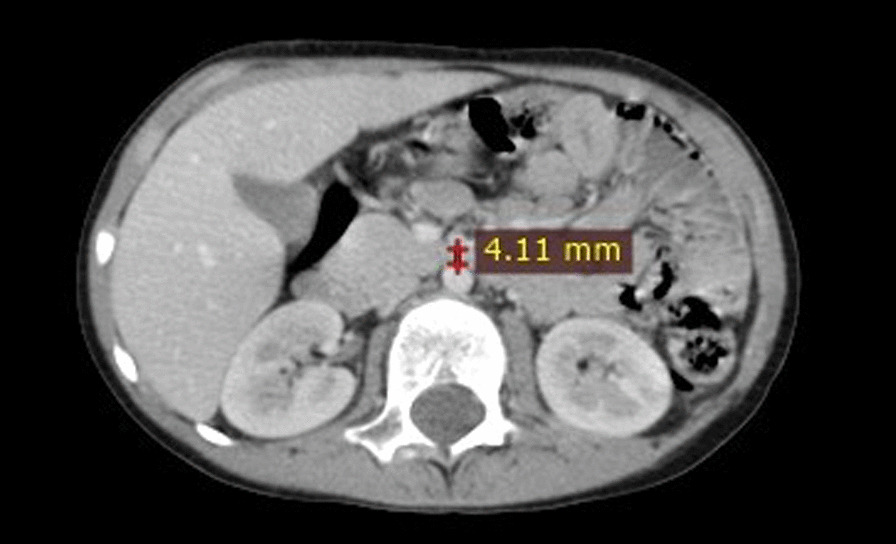
Contrast enhanced CT scan abdomen and pelvis (transverse view) showing the distance between the abdominal aorta and the superior mesenteric artery

An 8-year-old Arabian male patient was admitted to the gastrointestinal department at the Pediatric University Hospital in Damascus, Syria. He presented with abdominal pain with chronic periodic vomiting since he was two years old, occurring on average five to six times a month. Vomiting usually consists of partially digested food and occurs an hour postprandial. Previously, the patient's parents took him to several doctors who failed to find the right diagnosis. At the age of seven, he was diagnosed with appendicitis due to severe vomiting, acute abdominal pain, and fever. He was transferred to the hospital, where abdominal ultrasound (US) was performed but was inconclusive. The diagnosis relied on the clinical picture, and an appendectomy was performed. Unfortunately, After two months of persistent symptoms, he was diagnosed with an umbilical hernia and underwent another surgical operation, but he didn't improve. Intestinal malrotation was then suspected, and the patient underwent a gastrographin swallow, which showed a delay in the excretion of the gastrographin substance from the stomach to the small intestine, but no intestinal malrotation was found. Since the last seven months, the vomiting average has increased to approximately 10 to 15 times a month, and he was admitted to the hospital for three days. The case was diagnosed as a psychological problem with no treatment. Therefore the patient's parents decided to visit the Pediatric University Hospital where an accurate medical history and physical examination were conducted. His initial vital signs were normal, with no fever, Respiratory Rate (RR) of 18 breaths per minute, Heart Rate (HR) of 90 beats per minute, and no dehydration in the mucous membranes with a slight pale color. His weight is 22 kg, his height is 125 cm, and his body mass index (BMI) is 14.1, which falls in the Healthy Weight BMI category according to the body mass index-for-age percentiles: Boys, 2 to 20 years chart of Centers for Disease Control and Prevention (CDC). At this time, there was no vomiting or abdominal pain. In the examination of the skeleton, there was no significant malformation or skeletal pain. Inspection of the abdomen revealed two surgical scares one of them is above the umbilicus and the second in the right iliac fossa. Palpation of his abdomen was normal, with no pain, no abdominal masses, and no enlargement in the visceral organs was detected. Blood tests, including blood count (hemoglobin 128 g/L, white cell count 14.7 × 10^9^/L, and platelet count 607 × 10^9^/L), chemistries (sodium 137 mmol/L, potassium 5 mmol/L, blood urea nitrogen 29 mmol/L and serum creatinine 10 μmol/L), liver function tests (ALT 15 IU/L, HBsAg 0.315 and HCVAb 0.124) and inflammatory marker (CRP 0 mg/L), were normal. The urinalysis was within normal limits. The abdominal ultrasound showed no abnormalities. The upper gastrointestinal endoscopy showed a hiatal hernia measuring 3 cm. As a result, a contrasted CT scan of the abdomen and pelvis was performed, which revealed severe stenosis in the origin of the superior mesenteric artery (about 80%), an angle between the abdominal aorta and the superior mesenteric artery was approximately 13°(Fig. 1), and the distance between the aorta and the SMA was 4 mm (Fig. 2), which is lower than normal (10 to 20 mm). The clinical and imaging findings of the patient concluded the diagnosis of Superior mesenteric artery syndrome (SMAS). First, we considered conservative treatment, but since the patient was upset by the intensity of the symptoms and the parents were unable to complete this treatment, we decided to operate. During surgery, the third section of the duodenum was found to be under pressure from the upper mesenteric artery. Thus, the duodenum was released, and a side-to-side duodenojejunal anastomosis was made. The patient was put on a nasogastric tube for 5 days following surgery. Following that, oral feeding was introduced and well-tolerated, without any vomiting or abdominal pain, starting with liquids and progressing to solids. The patient was discharged from the hospital after a 10-day monitoring period, during which it was confirmed that his condition had improved and he was symptom-free. The patient was in good health after three months of follow-up, with no recurrence of symptoms.

## Discussion and conclusions

Superior mesenteric artery syndrome, also known as Wilkie's syndrome, is a rare vascular disease with a variable incidence ranging from 0.013 to 0.3%, and it is even rarer in children [1, 7]. While most SMAS cases occur in patients aged 10 to 39 years, with girls being more commonly affected than boys with a male–female ratio of 3:2 [2, 8], Our patient was an 8-year-old boy. The syndrome is usually associated with rapid and dramatic weight loss [9], which was not observed in our case. Although our patient had been vomiting since he was 2 years old, his weight was good and there was no medical history of rapid weight loss. The reason for the development of SMAS in children without weight loss is currently unexplained [5]. Another etiology that has been accused of causing this syndrome is spinal surgeries such as corrective surgery for scoliosis [10]. The clinical presentation of SMA syndrome is variable and nonspecific, including nausea, vomiting, abdominal pain, and weight loss [11]. Vomiting and abdominal pain are the prominent symptoms of our case which corresponds to previous studies [4]. Although these two symptoms are among the most common gastrointestinal symptoms and place the doctor in front of a plethora of differential diagnoses, few doctors include superior mesenteric artery syndrome among these. This could result in a delay in proper diagnosis and treatment. Other differential diagnoses that should be considered besides SMAS include intestinal malrotation, para-duodenal hernias, bezoars, pancreatitis, duodenitis, peptic ulcer disease, and Crohn's disease [10]. However, due to the rarity of the superior mesenteric artery syndrome, there is often a delay in diagnosis. The angle between the superior mesenteric artery and the abdominal aorta normally ranges from 38 to 56 degrees, when the angle is reduced to less than 25 degrees, SMAS is diagnosed [8]. We can determine the measurement of this angle using a contrasted CT scan. As a result, whenever a patient complains of vomiting and chronic abdominal pain, intestinal obstruction is suspected, common differential diagnoses were excluded, and the cause is anonymous, we should consider superior mesenteric artery syndrome. This syndrome may lead to several life-threatening complications, including dehydration, electrolyte imbalance, acute respiratory distress syndrome (ARDS) due to aspiration of gastric contents, severe duodenal dilatation, gastric perforations, and even death. Therefore, it is crucial to diagnose this syndrome as early as possible in order to avoid these complications [11]. Treatment for SMAS involves gaining weight in order to widen the aortomesenteric angle, and surgery is recommended when nonsurgical treatments are ineffective for symptomatic patients [12]. Since the parents of our patient did not have the financial ability to pursue conservative treatment and the child's symptoms were severe, we decided to perform the duodenojejunal anastomosis surgery.

## Data Availability

Not applicable.

## Abbreviation

SMAS: Superior mesentric artery syndrome
RR: Respiratory rate
HR: Heart rate
HBsAg: Hepatitis B surface antigen
HCVAb: Hepatitis C viral antibody
CRP: C reactive protein
US: Ultrasound
mm: Millimeter
cm: Centimeter
BMI: Body mass index
CDC: Centers for Disease Control and Prevention
ARDS: Acute respiratory distress syndrome
